# Ageing well in the urban environment: meeting the health and social needs of older Adults - study protocol for a prospective, longitudinal mixed-methods study

**DOI:** 10.1186/s12877-025-06185-0

**Published:** 2025-08-22

**Authors:** Marie Bolster, Abhijit Visaria, David Matchar, Denis Gerstorf, Tillman Schmitz, Benjamin Labohm, Raphael Kohl, Dagmar Haase, Paul Gellert, Angelique Chan, Wolfram J. Herrmann

**Affiliations:** 1 Institute of General Practice and Family Medicine, Charité - Universitätsmedizin Berlin, Berlin, Germany; 2 Centre for Ageing Research and Education, Duke-NUS Medical School, Singapore, Singapore; 3 Programme in Health Services and Systems Research, Duke-NUS Medical School, Singapore, Singapore; 4 Department of Medicine (General Internal Medicine) and Pathology, Duke Medical School, Durham, NC USA; 5 Department of Psychology, Humboldt-Universität zu Berlin, Berlin, Deutschland; 6 Socio-Economic Panel, Deutsches Institut für Wirtschaftsforschung (DIW), Berlin, Deutschland; 7 Geography Department, Humboldt-Universität zu Berlin, Berlin, Deutschland; 8 Institute of Medical Sociology and Rehabilitation Science, Charité - Universitätsmedizin, Berlin, Germany; 9 Department of Computational Landscape Ecology, Helmholtz Zentrum für Umweltforschung- UFZ, Leipzig, Deutschland

**Keywords:** Ageing, Cohort study, Study protocol, Urban health, Social determinants of health, Health geography, Health care

## Abstract

**Background:**

Urbanisation and ageing populations are global challenges for ageing well. This mixed-methods study examines the health and social needs of older adults in urban areas in Berlin, Germany and in Singapore, focusing on three key dimensions of healthy ageing and wellbeing: health, mobility, loneliness, and equity as a cross-cutting theme.

**Methods:**

The design follows a mixed-methods approach combining a quantitative longitudinal cohort study with qualitative interviews. For the quantitative cohort study, a standardised survey is carried out via home visits with a random sample of *n* = 1,050 adults aged 65 + years each in selected neighbourhoods in Berlin (three neighbourhoods) and Singapore (five neighbourhoods). The questionnaire comprises questions on health and health care utilization, neighbourhood, loneliness and social contacts, mobility and sociodemographic characteristics. Additionally, three functional tests (cognitive functioning, hand grip strength, and walking speed) are carried out with participants. Participants are followed-up after 12 months with a second survey interview. The survey data is linked with openly available geospatial data to allow for more in-depth study of the built-up and social environment and physical barriers in the selected neighbourhoods.

For the qualitative component, walking interviews are conducted with *n*=50 people each in Berlin and Singapore focusing on older adults’ subjective perspective on health, mobility, green spaces and loneliness in their urban neighbourhood. The results of the different study components will be triangulated.

**Discussion:**

The Ageing Well Study offers a valuable opportunity to assess the health and social needs of older adults in two distinct urban contexts. The mixed-methods design and utilization of multiple data sources enables an examination of the various dimensions of ageing well from disparate angles and perspectives. The extensive quantitative data set permits the examination of associations and interlinkages between the various dimensions of ageing well and the living environment, as well as the identification of changes over a 12-month period. By opting for home visits, participants with limited mobility are more likely to participate. Minor adaptations of the instruments and methods were implemented to best suit local context, administrative regulations, and feasibility at the study site.

**Trial registration:**

The study has been registered prospectively at the German Register for Clinical Studies (DRKS) (DRKS00033043, registration date: 16th Nov 2023) which is part of the WHO International Clinical Trials Registry Platform (WHO ICTRP).

## Background

Urbanisation and demographic change are global trends that pose challenges to ageing well. Germany and Singapore face similar macro-level trends albeit in different socio-cultural contexts. Both populations are rapidly ageing with the proportion of persons aged ≥ 65 years projected to be around one-third of the population in each country by 2050 [1]. Ageing is associated with an increase in chronic non-communicable diseases, as well as social and financial needs that in turn increase demands on health and social services [2, 3]. “Ageing well” occurs when “people can maintain satisfying and healthy lives as they age by making choices that optimize healthy, active, and safe living“ [4]. Given the increasing number of older adults in urban areas [5], this project focuses on three dimensions of ageing well: health, mobility, loneliness, and health equity as a cross-cutting theme in the urban settings of Berlin, Germany and Singapore.

The Ageing Well study is a collaborative study between the National University of Singapore (NUS), Charité - Universitätsmedizin Berlin, and the Humboldt-Universität zu Berlin (HU). It is funded with a grant from the Berlin University Alliance (BUA)-NUS Global Health Exploration Project.

### Dimension 1: health needs

An individual’s health status is shaped by a combination of factors, including medical conditions and social determinants. Older adults need services that reduce the risk of worsening health, transfer to assisted living, or death [6, 7]. An incongruence between having medical problems that require care, and receiving the necessary social and health care services can lead to unmet health needs of older persons. [8]. The Singapore Principal Investigators (PIs) in this project have developed the Simple Segmentation Tool (SST), a measurement scheme that identifies a hierarchy of health conditions combined with corresponding healthcare utilization that yields a categorization of the intensity and frequency of health needs [9]. The SST is an operationalisation of the ‘population segmentation’ approach to planning and evaluating health services and has been demonstrated to be predictive of worsening of health status, acute service use, and mortality [7, 10]. The BUA-NUS Global Health Initiative 2020-21 seed funding enabled the application of the SST to data from the Berlin Aging Study II so as to assess levels of unmet health and social needs. This preliminary work facilitated an expansion of the collaboration between BUA and NUS to situate health and social needs assessment within a broader framework, encompassing health needs, recreational/cooling greenspace, mobility, and loneliness.

### Dimension 2: mobility

Mobility is a central component of ageing well and mobility limitations affect about 35% of those aged 70 and most aged over 85 years [11]. Observational studies show mobility is connected to important factors for healthy and independent ageing, such as social support, walking speed, and a lower likelihood of falling due to minimizing barriers [11, 12]. Life-space mobility is defined as moving through geographically defined environments such as residential indoor and outdoor environments including green spaces [13–15]. It is related to physical health, but also includes social aspects that affect well-being, such as meaningful activities and social participation [16].

### Dimension 3: loneliness

Loneliness results from a perceived discrepancy between the desired and the realized quantity and quality of one’s social relationships [17–19], and is a key dimension of wellbeing at older ages [12, 20–22]. Loneliness is distinct from weak social ties [19] or depression [23]. At older ages, loneliness is associated with adverse health effects: depression, cognitive decline, functional limitations, chronic ailments, reduced healthcare utilization, and mortality [24–30]. Sociodemographic correlates of loneliness [30] as well as the relation between living environment and satisfaction, quality of life, and mental wellbeing are well studied [31–34]. In contrast, less is known about the roles of the living environment, neighbourhood cohesion, and the perception of an age-friendly environment for individual differences in loneliness.

### Cross-cutting theme: health equity

Health equity is generally assessed in terms of differential outcomes as a function of social determinants, such as socioeconomic status, and other features associated with ‘vulnerability’ such as gender, ethnicity, and age. The socio-economic status is known to impact wellbeing via multiple mechanisms, including differential access to basic service needs (i.e. housing, health care services, food), access to health and social services, and education. However, moderating factors have not been thoroughly studied yet [35, 36].

### Aims

The overall aim of the study is to examine associations between characteristics of the living environment of older adults (i.e., including health service availability and accessibility, built environment and recreational green space characteristics, and perceived age-friendliness) and three key dimensions of wellbeing, i.e., health, mobility, loneliness, and the crosscutting theme health equity. A secondary aim is to investigate changes across the different dimensions of wellbeing over 12 months.

## Methods/Design

### Study design

Ageing Well has a longitudinal mixed-methods design, comprising a cohort study and qualitative interviews. In nine neighbourhoods in Berlin and Singapore (three neighbourhoods in Berlin and five neighbourhoods in Singapore), we will conduct a two-wave cohort study of randomly chosen older adults as a survey using computer assisted personal interviews (CAPI). Participants will receive a 12-month follow-up (between 12 and 18 months after the initial interview). In addition to the cohort study, we carry out qualitative interviews with older adults including non-participatory observation of their use of built and greenspaces, as well as walking interviews conducted in outdoor and/or greenspace settings. These activities will be scheduled in the summer season to capture the effects of heat and hot days as health-impacting drivers. Data collection for the first wave of the cohort study in Berlin takes place from December 2023 onwards (first wave data collection: December 2023-June 2024, second wave data collection: December 2024 to July 2025, qualitative data collection: July to October 2024). In Singapore, data collection takes place February 2024 onwards.

### Sample size

The goal is to sample a total of *N* = 1,050 older adults each in Berlin and Singapore (i.e. *n* = 350 respondents in each of the three neighbourhoods in Berlin, *n* = 210 respondents in the five neighbourhoods in Singapore) for the cohort study, and *n* = 30 for the qualitative interviews. The differences between Berlin and Singapore in the number of neighbourhoods is due to differences in estimated response rates at baseline. Based on attrition rates in other longitudinal ageing studies, we estimate a 60% participation rate in the second wave [37, 38]. In Berlin, to boost our cross-sectional sample size, we will resample approximately 40% new participants in additional neighbourhoods in the second wave, who will only complete the first survey without a 12-month follow-up.

### Selection of neighbourhoods for data collection

#### Berlin

The city of Berlin is officially divided into 542 administrative neighbourhoods, known as LOR-planning areas (Life-space oriented planning areas), each with an average population of approximately 7,000 inhabitants [39]. Multiple datasets on these neighbourhoods are updated regularly and are made publicly available, providing insight into the proportion of older adults in each neighbourhood, their socio-economic status, and their migration background. In terms of public green spaces, land use, urban structure as well as structural density and population density, the Environmental Atlas of the city of Berlin provides extensive open-access datasets that serve as a starting point for our research.

Three neighbourhoods were selected for in-depth case studies based on a combination of official neighbourhood and environmental datasets, as well as our own previous research. The selection was made in collaboration with representatives from the local government and administration. The following criteria were considered for selection of neighbourhoods: (1) absolute number of older persons in the neighbourhood larger than sample size (minimum of 1,750 people over age 65), (2) medium to low socioeconomic status of the neighbourhood (3) variability in the built environment. Based on these criteria, three neighbourhoods in three districts were selected: (a) Tegel Süd (Reinickendorf), (b) Kurstraße + Eiswerder (Spandau, two adjacent planning areas were selected to fulfil minimum sample size requirements) and (c) Springpfuhl (Marzahn-Hellersdorf). For the resampling in the second data selection wave, the three additional adjacent neighbourhoods Alt Tegel (Reinickendorf), Auersbergstraße (Marzahn-Hellersdorf) and Ackerstraße (Spandau) were selected.

#### Singapore

In Singapore, studies of older adults have often focused on public housing residents and detailed the experiences of those living in more mature housing estates. A key contribution of this project will therefore be the inclusion of and comparison between participants in both public and private housing and mature and non-mature estates. The following criteria were considered for selecting the neighbourhoods for the study in Singapore: (1) a sizeable older adult population, (2) mix of relatively older and newer housing estates, i.e., those that developed in different decades since the 1950s to the mid-1990s. Five planning areas were chosen in Singapore: Tampines, Sengkang, Clementi, Ang Mo Kio and Kallang. Within each planning area, one sub-zone with a substantial proportion of older adults to the total population was selected. Planning areas, currently 55 in number, are used in Singapore primarily as development guide plan (DGP) areas, i.e., the level at which the statutory board, Urban Development Authority, draws up detailed land-use plans [40]. The study will obtain a random sample of 5,700 potential residential addresses across the sub-zones from the Singapore Department of Statistics. The sample frame from which addresses were chosen were residential addresses with at least one Singapore citizen or permanent resident aged 65 years and older. The distribution of addresses across housing types (i.e., government-built or Housing Development Board [HDB] 1–2 room apartments, 3-room apartments, 4-room apartments, 5-room or larger apartments) in the sample was proportional to the distribution of housing types in within each sub-zone.

### Selection of potential participants

#### Berlin

A random sample of 5,250 registered adults aged 65 or older in the four planning areas is drawn from the Berlin registration office. Participants receive an invitation letter explaining the study objectives, the participation process, the privacy policy, and a copy of the informed consent form. If more than one person per household is selected in the random sample, each receives an individual invitation to participate. Participants who do not wish to take part in the study can opt out by e-mail or telephone. Those who do not opt out receive a second letter with an appointment for the home visit, with another opportunity to opt out of the study or choose another interview date by telephone or e-mail. On the day of the home visit, participants are given the opportunity to participate then and there, to participate at another time, or to refuse to participate. Those who refuse are considered active refusals. Those who cannot be reached on the day of the announced site visit, receive at least one more letter with an announced home visit. Those who do not respond to any letters or home visits are considered passive refusals at the end of data collection. If participants agree to take part in the survey, the eligibility criteria (see Table 1) are checked. The same eligibility criteria apply for follow-up. Persons who move to a different neighbourhood between baseline and follow-up are only included if they move to a neighbourhood that is part of the study in the same district. All interviews are conducted in German.

**Table 1:** Inclusion and exclusion criteria for each study site

Inclusion criteria	Exclusion criteria
Min. 65 years of age (Berlin + Singapore), Max. 95 years of age (Singapore)	No written consent provided (Berlin + Singapore)
Registered resident in one of the selected neighbourhoods in Berlin/Self-reported non-temporary resident of an address in the Singapore sample	Cognitively not capable to understand the written consent form (Berlin)/Fewer than 5 correct answers on Abbreviated Mental Test (Singapore)
Written consent provided (Berlin + Singapore)	Linguistically unable to participate in the survey (Berlin + Singapore)
Citizen or permanent resident (Singapore)	Participant has a legal guardian (Berlin)
	Potential participant is unavailable or unable to participate because he/she is unable to hear, or unable to speak, or because of a chronic physical or mental health issue, or is admitted to an institution and cannot be interviewed during the study period (Singapore)

Written informed consent is obtained from all participants prior to the interview at both study sites.

#### Singapore

The inclusion criterion for participants in the study in Singapore first stipulates that a participant should be among the regular residents of the address in the sample (i.e., they should not be visitors or living at the address temporarily for any reason), be a Singapore citizen or permanent resident, and be aged 65–95 years. The specification of an upper age was mandated by the NUS Institutional Review Board.

During a visit to the address, an interviewer will first seek to prepare a list with the initials of all such individuals living at that address, by asking any adult household member with the exception of domestic helpers who are unlikely to provide this information to interviewers. At that time, the interviewer will ask if any of the listed older adults is unavailable or unable to answer the survey questionnaire, and if case of any affirmative responses ascertain if this is the case because he/she is (i) unable to hear, or unable to speak, or has a chronic physical or mental illness or disability which would prevent him/her from being able to respond to a survey questionnaire on their own, or (ii) currently admitted to a hospital, nursing home, hospice, or another institution for health or other reasons, and unlikely to be available for an interview during the study period. Such individuals are deleted from the list. At addresses where two or more older adults remain on the list, the interviewer will first use a random sequence generator (available gratis on https://www.random.org/) and assign each remaining individual in the list a serial number in the same sequence as generated. Then, the interviewer will use the true random number generator (on the same website) and specify the number of the older adults on the list. The random number generator will yield one of the serial numbers, and this individual is therefore the randomly selected potential participant. This process ensures that in addresses where more than one older adult is eligible and able to participate in the study, the selection of one potential participant is done at random.

Subsequently, the selected potential participant is administered a cognitive status test. If the selected potential participant answers five or more questions correctly from the ten-item Abbreviated Mental Test– Singapore, a criterion that has been used previously in surveys administered with community-dwelling older adults [41], he/she is deemed eligible to continue [42].

If a selected potential participant refuses to participate in the process outlined above or subsequently expresses unwillingness to participate in the study, a replacement potential participant is not chosen. Similarly, if an adult household member refuses to provide the initial information that allows interviewers to list eligible older adult residents at an address or disallows the interviewer from speaking to or contacting the selected potential participant, the study deems that household as having refused participation in the study. For reasons of propriety, the interviewer will not seek to speak to another adult household member at that address or visit it again. The interviews are conducted in one of the four official languages English, Mandarin, Malay and Tamil, depending on the participants preferences and abilities.

### Data collection instruments

Data collection is conducted as a computer-assisted personal interview (CAPI) at the participants‘ home. There will be a baseline data collection and a follow-up data collection after twelve months (earliest 12 months and latest 18 months after the initial interview). A standardized questionnaire has been developed, with minor adaptations to best suit local context and feasibility at the study site. The following instruments are used to assess the different dimensions of ageing as well as potential outcomes and risk factors (Table 2).

**Table 2:** Items in the questionnaire ageing well, by study site and data collection wave

Dimension	Assessed Variables	Instrument	Baseline		Follow-up	
			Berlin	Singapore	Berlin	Singapore
*Health Needs*	Self-rated Health Status (overall, vision, hearing)		x	x	x	x
	Frailty	Frailty Criteria according to Fried	x	x	x	x
	Mental health	PHQ-4	x	x	x	x
	Health care utilization	Simple Segmentation Tool (SST) & European Health Interview Survey (EHIS)	x	x	x	x
	Primary Care continuity	Continuity of care	x	x	x	x
	Diagnosed health conditions	SST	x	x	x	x
	Activation of Care	SST	x	x	x	x
	Medication	SST	x	x	x	x
	Tobacco consumption	GEDA/EHIS	x		x	
*Loneliness & wellbeing*	Loneliness	UCLA Loneliness short scale (locally adapted)	x	x	x	x
	Risk of social isolation	Lubben Social Network Short Scale − 6	x	x	x	x
	Control Beliefs	Locus of Control	x		x	
	Resilience	Brief Resilience Scale		x		x
	Life satisfaction		x	x	x	x
*Mobility*	Mobility at home and in the community	University of Alabama at Birmingham Life Space Assessment (UAB-LSA)	x	x	x	x
	Functional Assessment: Daily activities	SST & GALI Score	x	x	x	x
	Use of assistive devices (Mobility)	SST	x	x	x	x
	Physical activity	International Physical Activity Questionnaire- Short form (IPAC-SF)	x	x	x	x
*Living environment*	Type of housing		x	x		
	Duration living in current housing		x	x		
	Neighbourhood Social cohesion		x	x	x	
	Environmental Age-friendliness	Age- friendly Environmental Assessment Tool (AFEAT)	x	x	x	x
	Access to green spaces	In AFEAT format	x	x	x	x
*Sociodemographic characteristics*	Household size		x	x	x	x
	Marital status		x	x	x	x
	Number of living children		x	x	x	x
	Gender		x	x		
	Migration background		x	x		
	East/west Germany background		x			
	Educational background		x	x	x	x
	SES	MacArthur Scale (Subjective social status)	x	x	x	
	Sufficient income		x	x	x	
*Functional Assessments*	Cognitive Status	Digit Symbol Substitution Test	x	x	x	x
	Cognitive Screener			x		x
	Handgrip strength	Dynamometer measurement	x	x	x	x
	Walking speed	Walking speed over 2.5 m	x	x	x	x

### Health needs

To identify health needs of older adults, we assess health status, diagnosed health conditions, frailty, health care utilization, continuity of primary care, activation of care, medication and tobacco consumption.

#### Health status

Participants are asked to rate their health status (general health, vision, and hearing) using five categories (“excellent”, “very good”, “good”, “fair” and “poor”). For questions regarding vision and hearing, two additional categories are included (“loss of vision (no vision) in both eyes” and “not able to hear in both ears”).

In addition, we assess self-reported medical professional-diagnosed health conditions (over the lifetime) as well as disease-specific health care utilization (past 6 months) using questions from the community survey version of the SST [10]. The ever-diagnosed health conditions are classified into potentially severe or life-threatening conditions (e.g., cancer, heart attack, cerebrovascular disease such as stroke, chronic respiratory illness) and others (e.g., hypertension, arthritis, chronic back pain). This information is used in combination with healthcare utilizations for these conditions and the Global Activity Limitation Indicator (described below) to segment participants into different global impression of health categories.

#### Mental health status

We assess the presence of symptoms of anxiety and/or depression using the PHQ-4 (Peoples Health Questionnaire-4). The PHQ-4 is an established, validated 4-item screening tool for depression and anxiety and is highly correlated with disability days, functional impairment and health care use [43, 44]. It includes two-items on depressive symptoms and two-items on symptoms of anxiety.

#### Frailty

Frailty is a clinical syndrome that is predictive of a range of health outcomes in older adults, including falls, hospitalizations, and death [45]. Frailty is determined according to Fried´s Frailty phenotype, which is defined by the presence of at least three of the following criteria: unintentional weight loss (> 5 kg in the past 12 months), weak grip strength, slow walking speed, self-reported exhaustion and low physical activity [45], measured using the Short -Form of the International Physical Activity questionnaire (IPAC-SF).

#### Health care utilization

Questions from the German version of the European Health Interview Survey (GEDA/EHIS 2014/2015) [46] are used to assess the type of insurance, number of consultations with a General Practitioner (last 12 months), number of consultations with a specialist (last 12 months), visits to the emergency room (last 6 months), and overnight hospital stays (last 12 months). Additionally, we assess disease-specific health care utilisation (past 6 months) as part of the SST.

#### Primary care continuity

Primary care continuity is related to a range of health outcomes including reduced mortality, lower health care costs, and hospitalisations [47, 48]. We use items on longitudinal continuity of care (care site and provider duration) from a continuity of care model used in the National Health and Health Services Use Questionnaire (NHHSUQ) [49]. The items assess the most frequently visited health care site, duration of care at care site, presence of a specific care provider, duration of care from care provider and changes in care provider in the last 12 months.

#### Patient activation of care

Patient activation refers to the knowledge, confidence, skills, and abilities of patients to manage their own care. Patient activation is a known predictor for a range of health and treatments outcomes, health behaviours, as well as lower hospitalization rates and visits to A&E [50]. Patient activation is assessed using three items as used in the SST: patient perceived knowledge about their own condition (“very little”, “something”, “a lot”), confidence in management of own physical ailments (“not at all confident”, “a little confident”, “somewhat confident”, “quite confident”, “extremely confident”), and problems in understanding information about their own health (“always”, “often”, “sometimes”, “occasionally”, “never”) [10].

#### Use of medication

As many older patients have multimorbid health needs and require polypharmacy, we ask for the use and number of prescription drugs taken regularly.

#### Smoking behaviour

As smoking is a risk factors for many health outcomes, tobacco use is evaluated using three questions from the GEDA/EHIS 2014/15 questionnaire [46] on current use of tobacco products, past tobacco use and total duration of tobacco use in years.

### Loneliness & wellbeing

#### Loneliness

Loneliness is linked to outcomes in mental health, general health, well-being, physical health, sleep and cognition [51]. We use adapted and abbreviated versions of the UCLA Loneliness Scale [52]. We employ an 8-item version, adapted from the revised UCLA Scale (Version II), with a 5-point response scale, to allow for comparison with other ageing studies (BASE II Berlin Ageing Study) [53].

#### Risk of social isolation

The Lubben Social Network Short Scale is used as a screening tool to identify persons at risk of social isolation. The six-item scale assesses social ties with family and friends and has been used and validated in older populations in a European and Singaporean context [54–56]. Social Isolation has been linked to higher mortality among other health outcomes [57, 58].

#### Control beliefs (Berlin survey only) & resilience (Singapore survey only)

Both control beliefs and resilience are associated with wellbeing in older age [59, 60]. We use three items on internal locus of control as used in the BASE II study in Germany [61]. Participants are asked how much the following three statements apply to them on a scale of 1 = not at all applicable to 5 = very much applicable: “I can determine the good things that are happening in my life”, “If I get what I want, it is usually because I have done a lot for it myself”, “I can influence the good and beautiful things in my life myself”.

In Singapore, resilience is measured using the Brief Resilience scale (BRS) [62]. The six-item BRS asks participants to what extent they agree (five response options from strongly agree, disagree, neutral, to agree and strongly agree) with statements about bouncing back after hard times, making it through and recovering from or getting over from stressful events.

#### General life satisfaction

To determine general life satisfaction participants are asked: “Overall, how satisfied are you currently with your life?” and given a 10-point response scale. 0 = completely unsatisfied and 10 = completely satisfied [63].

### Mobility

#### Life space assessment

The University of Alabama at Birmingham Study of Aging Life-Space Assessment (UAB-LSA) measures mobility of older adults in their home and community [64]. It has been validated and is associated with a range of outcomes, including limitations in activities and instrumental activities of daily living, functional mobility and self-rated health [15, 16].

#### Functional assessment: daily activities, assistive devices & Gali score

We measured functional capacity among participants by assessing the extent to which health- or physical limitations affected the ability to independently perform different everyday activities (Basic and Instrumental Activities of Daily Living). The GALI Score (Global Activity Limitation Index) [65] is used to determine the overall (6 month) limitations in everyday activities due to health conditions. The presence of limitations reported on the GALI are used for population segmentation as per the SST framework, for example, in classifying participants with chronic conditions as having symptoms that interfere with or restrict usual function even if they are not necessarily hospitalized for that condition.

### Living environment

#### Type of housing and duration of living in current housing

Participants are asked in what type of housing they live (e.g. rented, owned, apartment, etc.). The answer choices are adapted to the local Singapore/Berlin housing context. Participants are then asked how long they have approximately lived in the current housing (in years/months).

#### Neighbourhood social cohesion

Self-perceived neighbourhood cohesion is measured using 7-items, which have been used in the Health and Retirement study (HRS) [66]. Participants are asked to rate the 7 statements about the neighbourhood regarding the community, trustworthiness, safety, cleanliness, friendliness, helpfulness, attractiveness using five answer categories (“strongly disagree, disagree, neither agree/disagree, agree, strongly agree”). Self-perceived neighbourhood social cohesion has been found to be associated with a lower risk for a range of physical and mental health outcomes, e.g. myocardial infarction, falls, anxiety and depression [67–69].

#### Environmental age-friendliness

Age-friendliness of the environment is a strong predictor for quality of life and loneliness in older adults [70]. The Age-friendly environment Assessment Tool (AFEAT) is a reliable, validated instrument, that has been developed based on the WHO age-friendly environment checklist [70]. It assesses individual perceptions of the individual environment (e.g. accessibility and availability of services, medical care, public transportation etc.) using 10-items on a 5-point Likert scale [70]. We use an adapted version of the AFEAT questionnaire with some additional items. In Singapore, the module is expanded, with the aim of collecting data on the perception of additional context-specific services: e.g., whether neighbourhood centres such as Active Ageing Centres (a major public policy initiative aimed at older adults in Singapore) had activities and events of interest to participants, or the ease with which participants are able to read/understand information about programmes and services on posters, flyers, notice boards, etc.

#### Access to green spaces

To determine subjective access to green spaces, a 5-point Likert scale statement (“I live close to public green spaces such as parcs, gardens or woodlands”) is added in the same format as the AFEAT items.

### Socioeconomic measurements

To assess sociodemographic variables a range of standardized questions are used. The following socioeconomic characteristics are measured: gender, household size, marital status, number of living children, native language, migration background (Berlin only), ethnicity (Singapore only), East/west German background (Germany only), educational attainment, income sufficiency, and housing type (Singapore only; housing type has been found in previous research to be proportional to household income and frequently used as a proxy for household socioeconomic status [71]). In the German version the majority of questions are based on the Diversity Minimal Item Set (DiMIS) [72].

The MacArthur Scale of Subjective Social Status [73] is selected as a measure for individual socioeconomic status (SES) as it highly correlated with more objective multidimensional SES measures, that tend to yield high levels of missing values in surveys, e.g. on questions on income [74].

### Functional assessments

#### Cognitive status

Cognitive status is assessed using a paper-and-pencil digit symbol substitution test (DSST). The DDST is a valid and established instrument for cognitive disfunction, that is quick to implement [75]. DDST is a strong predictor for mortality and functional outcomes [75, 76].

#### Hand grip strength and walking speed

Hand grip strength and walking speed are assessed as part of the criteria for frailty. Unless any counterindications are present, the participant’s hand grip strength is determined twice for each hand using a standardized dynamometer. For walking speed, the time it takes participants to cover a marked distance of 2.5 (using a walking aid if necessary) is stopped. The walking speed measurements are taken at a clear and level space (uncarpeted, if possible) of 2.5 m at the participants homes. Start and end points are measured out by the interviewer and marked with masking tape. Measurements are taken twice and the average of the two times is taken as a participant’s walking speed.

### Other data sources

The survey data will be merged with openly available geospatial data from sources such as Open Street Map (OSM), or datasets from the Berlin Senate’s Geodata Portal. Among the data used will be Points-of-interest from OSM, which will be filtered and classified into categories relevant to the elderly. Additionally, we will model walkability of the target neighbourhoods with a focus on recreational use of urban green spaces (UGS), using two indices, namely the local significance (LS) and detour index (DI). LS will be used as to model recreational flows and potential overcrowding effects of UGS, while DI will serve as a proxy for barriers that elderly people may encounter on their ways to UGS. All data will be modelled in 300 m, 500 m and 1000 m network distances around all residential buildings in the target LORs and later matched via address to the survey data to fulfil data protection requirements.

## Qualitative data collection and analysis

We will conduct and analyse *n* = 50 qualitative walking interviews with older adults to understand their usage patterns, activities and perceptions of physical and other barriers encountered in urban green spaces and streets.

First, the Berlin team will prepare the qualitative interview guidelines and share the guidelines with the Singapore team. The interview guidelines will be tested. We aim to randomly select participants from contrasting neighbourhoods to gain a diverse range of perspectives. Whenever possible and always in agreement with the persons approached, we intend to conduct these interviews as walking interviews through the study site alongside our interviewees on a route chosen individually by the participants according to their preferences, adapting our approach based on their mobility. Interviews will be transcribed and pseudonymised. Interview transcript will be analysed qualitative with a coding process oriented towards the grounded theory paradigm.

## Quantitative data analysis

After data cleaning, we will conduct both cross-sectional and longitudinal analysis of the data. We will first examine missing values and if there is a need for multiple imputation. We will then conduct descriptive data analyses and regression analyses taking the multilevel type of the data into account. Sensitivity analyses will be conducted to ensure the robustness of our results.

## Triangulation of results

We will triangulate the quantitative and qualitative results on neighbourhood level and a general level. In joint triangulation workshops researchers will bring their results together discussing the results and topics from an interdisciplinary mixed methods perspective.

## Discussion

### Strengths and limitations of the study

The mixed-methods design, which incorporates multiple study sites and data sources, enables an examination of the various dimensions of ageing well from a range of perspectives. The large sample survey provides a substantial data set for quantitative analysis and can be linked to objective geographical geographic indicators at the neighbourhood or street level. The large dataset allows for pooled analyses of the sample using multilevel analysis to account for differences in the study sites as well as comparative or stratified analyses, where deemed relevant, to identify differences between or within the subsamples. As the samples are drawn at random from the registry offices at both study sites, the pool of potential candidates reflect the populations aged 65 or older of the respective neighbourhoods in terms of the socioeconomic characteristics including gender, age distribution and ethnic or cultural diversity. By opting for home visits (including visits in nursing homes), older people with reduced mobility are more likely to participate and the burden of participation is reduced. Furthermore, the qualitative data offer a more subjective insight into the needs of individuals and their perceptions of their neighbourhoods, which may not be fully captured in the standardised interviews.

The longitudinal design can capture short-term changes in the participants health and social needs within 12 months. The study has the potential to be expanded into an ongoing longitudinal study with multiple follow-ups in the long term.

The study also comes with several methodological limitations. Due to the differences in context and administrative regulations, the methodology and questionnaires had to be adapted slightly between Singapore and Berlin with regards to sampling, data collection and sociodemographic measures. Secondly, individuals who are advanced in age, experience significant health limitations, or express reservations about a home visit are less likely to participate in the study. Additionally, at the Berlin study site, the interviews are conducted in German, which limits participation from those who do not have sufficient German skills to complete the interview.

The selection of three to five neighbourhoods in each city may limit the generalisability of the results, however, the use of many standardised instruments allows the results to be compared with other cohort studies of ageing.

## Data Availability

No datasets were generated or analysed during the current study.

## Abbreviations

A&E: Accidents and Emergency Department
AFEAT: Age Friendly Environment Assessment Tool
BASE II: Berlin Ageing Study II
BUA: Berlin University Alliance
BRS: Brief Resilience Scale
CAPI: Computer Assisted Personal Interviews
DI: Detour Index
DSST: Digital Symbol Substitution Test
DiMIS: Diversity Minimal Item Set
DRKS: Deutsches Register für Klinische Studien
EHIS: European Health Interview Survey
GALI: Global Activity Limitation Index
GEDA: Gesundheit in Deutschland Aktuell (German Version of the European Health Interview survey)
HDB: Housing Development Board
HRS: Health and Retirement Study
HU: Humboldt–Universität zu Berlin
IPAC-SF: Short form of the International Physical Activity Questionnaire
LS: Local Significance
LOR: Life Space Oriented Areas
NHHSUQ: National Health and Health Services Use Questionnaire
NUS: National University of Singapore
OSM: Open Street Map
PHQ - 4: People’s Health Questionnaire − 4
PIs: Principal Investigators
SES: Socio–economic Status
SST: Simple Segmentation Tool
UAB-LSA: University of Alabama at Birmingham Study of Aging Life–Space Assessment
UGS: Urban green spaces
UCLA: University of California, Los Angeles

## References

[1] United Nations Department of Economic and Social Affairs, Population Division. World Population Prospects 2019, Volume II: Demographic Profiles (ST/ESA/SER.A/427). New York: United Nations; 2019. https://www.un.org/development/desa/pd/sites/www.un.org.development.desa.pd/files/un_2019_wpp_vol2_demographic-profiles.pdf

[2] Prince MJ, Wu F, Guo Y, Gutierrez Robledo LM, O’Donnell M, Sullivan R, et al. The burden of disease in older people and implications for health policy and practice. Lancet. 2015;385(9967):549–62. 25468153 10.1016/S0140-6736(14)61347-7

[3] Singapore Ministry of Health, Institute for Health Metrics and Evaluation. The Burden of Disease in Singapore, 1990–2017: An Overview of the Global Burden of Disease Study 2017 Results. Seattle, WA: Institute for Health Metrics and Evaluation; 2019. https://www.healthdata.org/sites/default/files/files/policy_report/2019/GBD_2017_Singapore_Report.pdf.

[4] Hawkins BA. Aging well: toward a way of life for all people. Prev Chronic Dis. 2005;2(3):A03. 15963305 PMC1364512

[5] United Nations Department of Economic and Social Affairs, Population Division. World Urbanization Prospects: The 2018 Revision (ST/ESA/SER.A/420). New York: United Nations; 2019. https://population.un.org/wup/assets/WUP2018-Report.pdf.

[6] Chong JL, Matchar DB. Benefits of Population Segmentation Analysis for Developing Health Policy to Promote Patient-Centred Care. Ann Acad Med Singap. 2017;46(7):287–9. 28821893

[7] Chong JL, Matchar DB, Tan Y, Sri Kumaran S, Gandhi M, Ong MEH, et al. Population Segmentation Based on Healthcare Needs: Validation of a Brief Clinician-Administered Tool. J Gen Intern Med. 2021;36(1):9–16. 32607929 10.1007/s11606-020-05962-4 PMC7859147

[8] Allin S, Grignon M, Le Grand J. Subjective unmet need and utilization of health care services in Canada: What are the equity implications? Social Science & Medicine. 2010;70(3):465–72. 19914759 10.1016/j.socscimed.2009.10.027

[9] Chong JL. Matching Health and Social Services to Needs in an Ageing Population: Development of the Simple Segmentation Tool. Singapore: Duke-NUS Medical School; 2018. https://scholarbank.nus.edu.sg/entities/publication/09710c87-48c8-4f9c-b44e-b99d95d744c2.

[10] Chong JL, Low LL, Matchar DB, Malhotra R, Lee KH, Thumboo J, et al. Do healthcare needs-based population segments predict outcomes among the elderly? Findings from a prospective cohort study in an urbanized low-income community. BMC Geriatrics. 2020;20(1):78. 32103728 10.1186/s12877-020-1480-9 PMC7045405

[11] Freiberger E, Sieber CC, Kob R. Mobility in Older Community-Dwelling Persons: A Narrative Review. Front Physiol. 2020;11:881. 33041836 10.3389/fphys.2020.00881 PMC7522521

[12] Luo Y, Hawkley LC, Waite LJ, Cacioppo JT. Loneliness, health, and mortality in old age: a national longitudinal study. Soc Sci Med. 2012;74(6):907–14. 22326307 10.1016/j.socscimed.2011.11.028 PMC3303190

[13] Baker PS, Bodner EV, Allman RM. Measuring life-space mobility in community-dwelling older adults. J Am Geriatr Soc. 2003;51(11):1610–4. 14687391 10.1046/j.1532-5415.2003.51512.x

[14] Johnson J, Rodriguez MA, Al Snih S. Life-Space Mobility in the Elderly: Current Perspectives. Clin Interv Aging. 2020;15:1665–74. 32982200 10.2147/CIA.S196944 PMC7501960

[15] Ullrich P, Werner C, Abel B, Hummel M, Bauer JM, Hauer K. Assessing life-space mobility: A systematic review of questionnaires and their psychometric properties. Z Gerontol Geriatr. 2022;55(8):660–6. 35244765 10.1007/s00391-022-02035-5 PMC9726808

[16] Mümken SA, Gellert P, Stollwerck M, O’Sullivan JL, Kiselev J. Validation of the German Life-Space Assessment (LSA-D): cross-sectional validation study in urban and rural community-dwelling older adults. BMJ Open. 2021;11(7):e049926. 34230022 10.1136/bmjopen-2021-049926 PMC8261868

[17] De Jong Gierveld J, Van Tilburg T. The De Jong Gierveld short scales for emotional and social loneliness: tested on data from 7 countries in the UN generations and gender surveys. Eur J Ageing. 2010;7(2):121–30. 20730083 10.1007/s10433-010-0144-6 PMC2921057

[18] Peplau LA, Perlman D. Loneliness: A sourcebook of current theory, research, and therapy: John Wiley & Sons Inc; 1982.

[19] Hawkley LC, Hughes ME, Waite LJ, Masi CM, Thisted RA, Cacioppo JT. From Social Structural Factors to Perceptions of Relationship Quality and Loneliness: The Chicago Health, Aging, and Social Relations Study. The Journals of Gerontology: Series B. 2008;63(6):S375–84. 10.1093/geronb/63.6.s375 PMC2769562 19092047

[20] Cacioppo JT, Cacioppo S. The growing problem of loneliness. Lancet. 2018;391(10119):426. 29407030 10.1016/S0140-6736(18)30142-9 PMC6530780

[21] Cacioppo JT, Hawkley LC, Crawford LE, Ernst JM, Burleson MH, Kowalewski RB, et al. Loneliness and health: Potential mechanisms. Psychosomatic Medicine. 2002;64(3):407–17. 12021415 10.1097/00006842-200205000-00005

[22] De Jong Gierveld J. A review of loneliness: concept and definitions, determinants and consequences. Reviews in Clinical Gerontology. 1998;8(1):73–80.

[23] Cacioppo JT, Hughes ME, Waite LJ, Hawkley LC, Thisted RA. Loneliness as a specific risk factor for depressive symptoms: cross-sectional and longitudinal analyses. Psychol Aging. 2006;21(1):140–51. 16594799 10.1037/0882-7974.21.1.140

[24] Ge L, Yap CW, Ong R, Heng BH. Social isolation, loneliness and their relationships with depressive symptoms: A population-based study. PLoS One. 2017;12(8):e0182145. 28832594 10.1371/journal.pone.0182145 PMC5568112

[25] Wilson RS, Krueger KR, Arnold SE, Schneider JA, Kelly JF, Barnes LL, et al. Loneliness and risk of Alzheimer disease. Archives of general psychiatry. 2007;64(2):234–40. 17283291 10.1001/archpsyc.64.2.234

[26] Malhotra R, Tareque MI, Saito Y, Ma S, Chiu C-T, Chan A. Loneliness and health expectancy among older adults: A longitudinal population-based study. Journal of the American Geriatrics Society. 2021;69(11):3092–102. 34231876 10.1111/jgs.17343

[27] Cacioppo JT, Hawkley LC, Thisted RA. Perceived social isolation makes me sad: 5-year cross-lagged analyses of loneliness and depressive symptomatology in the Chicago Health, Aging, and Social Relations Study. Psychol Aging. 2010;25(2):453–63. 20545429 10.1037/a0017216 PMC2922929

[28] Lim KK, Chan A. Association of loneliness and healthcare utilization among older adults in Singapore. Geriatrics & Gerontology International. 2017;17(11):1789–98. 28060447 10.1111/ggi.12962

[29] Chan A, Raman P, Ma S, Malhotra R. Loneliness and all-cause mortality in community-dwelling elderly Singaporeans. Demographic Res. 2015;S15(49):1361–82.

[30] Raymo JM, Wang J. Loneliness at Older Ages in the United States: Lonely Life Expectancy and the Role of Loneliness in Health Disparities. Demography. 2022;59(3):921–47. 35502830 10.1215/00703370-9937606

[31] Liu Y, Dijst M, Faber J, Geertman S, Cui C. Healthy urban living: Residential environment and health of older adults in Shanghai. Health & Place. 2017;47:80–9. 28778036 10.1016/j.healthplace.2017.07.007

[32] Toma A, Hamer M, Shankar A. Associations between neighborhood perceptions and mental well-being among older adults. Health & Place. 2015;34:46–53. 25909762 10.1016/j.healthplace.2015.03.014

[33] Zhang W, Liu S, Zhang K, Wu B. Neighborhood Social Cohesion, Resilience, and Psychological Well-Being Among Chinese Older Adults in Hawai’i. Gerontologist. 2020;60(2):229–38. 31322675 10.1093/geront/gnz104

[34] Elliott J, Gale CR, Parsons S, Kuh D. Neighbourhood cohesion and mental wellbeing among older adults: A mixed methods approach. Social Science & Medicine. 2014;107:44–51. 24602970 10.1016/j.socscimed.2014.02.027

[35] Montano D. Socioeconomic status, well-being and mortality: a comprehensive life course analysis of panel data, Germany, 1984–2016. Archives of Public Health. 2021;79(1):40. 33762017 10.1186/s13690-021-00559-7 PMC7992831

[36] Yang F, Pang JS. Socioeconomic status, frailty, and subjective well-being: A moderated mediation analysis in elderly Chinese. J Health Psych. 2016;23(7):961–70. 10.1177/1359105316675211 27821682

[37] Lara E, Miret M, Olaya B, Caballero FF, Morillo D, Moneta MV, et al. Cohort Profile: The Spanish Longitudinal Study on Ageing and Health (Edad Con Salud). Int J Epidemiology. 2022;51(4):e189–99. 10.1093/ije/dyac118 35712861

[38] Kawai H, Ejiri M, Tsuruta H, Masui Y, Watanabe Y, Hirano H, et al. Factors associated with follow-up difficulty in longitudinal studies involving community-dwelling older adults. PLOS ONE. 2020;15(8):e0237166. 32745148 10.1371/journal.pone.0237166 PMC7398506

[39] Senatsverwaltung für Stadtentwicklung BuW. Lebensweltlich orientierte Räume (LOR) in Berlin 2025 [Available from: https://www.berlin.de/sen/sbw/stadtdaten/stadtwissen/sozialraumorientierte-planungsgrundlagen/lebensweltlich-orientierte-raeume/.

[40] National Library Board of Singapore. Development guide plans 2021 [Available from: https://www.nlb.gov.sg/main/article-detail?cmsuuid=0b508b32-e1b8-487f-b8da-b3baa739a514.

[41] Chan A MR, Manap NB, Ting YY, Visaria A, Cheng GH-L, Goh VSM, Tay PKC, Lee JML, Maulod A,. Transitions in Health, Employment, Social Engagement And Intergenerational Transfers In Singapore Study (THE SIGNS Study)– I: Descriptive Statistics and Analysis of Key Aspects of Successful Ageing. Singapore: Centre for Ageing Research and Education, Duke-NUS Medical School; 2018. https://www.duke-nus.edu.sg/docs/librariesprovider3/research-policy-brief-docs/the-signs-study---i-report.pdf.

[42] Sahadevan S, Lim PP, Tan NJ, Chan SP. Diagnostic performance of two mental status tests in the older chinese: influence of education and age on cut-off values. Int J Geriatr Psychiatry. 2000;15(3):234–41. 10713581 10.1002/(sici)1099-1166(200003)15:3<234::aid-gps99>3.0.co;2-g

[43] Löwe B, Wahl I, Rose M, Spitzer C, Glaesmer H, Wingenfeld K, et al. A 4-item measure of depression and anxiety: validation and standardization of the Patient Health Questionnaire-4 (PHQ-4) in the general population. J Affect Disord. 2010;122(1–2):86–95. 19616305 10.1016/j.jad.2009.06.019

[44] Kroenke K, Spitzer RL, Williams JB, Löwe B. An ultra-brief screening scale for anxiety and depression: the PHQ-4. Psychosomatics. 2009;50(6):613–21. 19996233 10.1176/appi.psy.50.6.613

[45] Fried LP, Tangen CM, Walston J, Newman AB, Hirsch C, Gottdiener J, et al. Frailty in older adults: evidence for a phenotype. J Gerontol A Biol Sci Med Sci. 2001;56(3):M146-56. 11253156 10.1093/gerona/56.3.m146

[46] Koch-Institute Robert. Fragebogen zur Studie „Gesundheit in Deutschland aktuell“: GEDA 2014/2015-EHIS. J Health Monitoring. 2017;2(1):105–35.

[47] Bazemore A, Petterson S, Peterson LE, Bruno R, Chung Y, Phillips RL Jr. Higher Primary Care Physician Continuity is Associated With Lower Costs and Hospitalizations. Ann Fam Med. 2018;16(6):492–7. 30420363 10.1370/afm.2308 PMC6231930

[48] Baker R, Freeman GK, Haggerty JL, Bankart MJ, Nockels KH. Primary medical care continuity and patient mortality: a systematic review. British J Gen Pract. 2020;70(698):e600–11. 10.3399/bjgp20X712289 PMC7425204 32784220

[49] Bentler SE, Morgan RO, Virnig BA, Wolinsky FD. Evaluation of a patient-reported continuity of care model for older adults. Quality Life Res. 2014;23(1):185–93. 10.1007/s11136-013-0472-z 23868458

[50] Hibbard J, Gilburt H. Supporting people to manage their health - An introduction to patient activation. The Kings Fund; 2014 May 2014. https://assets.kingsfund.org.uk/f/256914/x/d5fbab2178/supporting_people_manage_their_health_2014.pdf.

[51] Park C, Majeed A, Gill H, Tamura J, Ho RC, Mansur RB, et al. The Effect of Loneliness on Distinct Health Outcomes: A Comprehensive Review and Meta-Analysis. Psychiatry Res. 2020;294. 10.1016/j.psychres.2020.113514 33130511

[52] Russell D, Peplau LA, Cutrona CE. The revised UCLA Loneliness Scale: concurrent and discriminant validity evidence. J Pers Soc Psychol. 1980;39(3):472–80. 7431205 10.1037//0022-3514.39.3.472

[53] Suanet B, Drewelies J, Duezel S, Eibich P, Demuth I, Steinhagen-Thiessen E, et al. Historical change in trajectories of loneliness in old age: Older adults today are less lonely, but do not differ in their age trajectories. Psychol Aging. 2024;39(4):350–63. 38900502 10.1037/pag0000803

[54] Merchant RA, Liu SG, Lim JY, Fu X, Chan YH. Factors associated with social isolation in community-dwelling older adults: a cross-sectional study. Qual Life Res. 2020;29(9):2375–81. 32253669 10.1007/s11136-020-02493-7

[55] Lubben J, Blozik E, Gillmann G, Iliffe S, von Renteln Kruse W, Beck JC, et al. Performance of an Abbreviated Version of the Lubben Social Network Scale Among Three European Community-Dwelling Older Adult Populations. Gerontologist. 2006;46(4):503–13. 16921004 10.1093/geront/46.4.503

[56] Ge L, Yap CW, Heng BH. Associations of social isolation, social participation, and loneliness with frailty in older adults in Singapore: a panel data analysis. BMC Geriatr. 2022;22(1):26. 34991493 10.1186/s12877-021-02745-2 PMC8734362

[57] Wang F, Gao Y, Han Z, Yu Y, Long Z, Jiang X, et al. A systematic review and meta-analysis of 90 cohort studies of social isolation, loneliness and mortality. Nat Hum Behav. 2023;7(8):1307–19. 37337095 10.1038/s41562-023-01617-6

[58] Holt-Lunstad J, Steptoe A. Social isolation: An underappreciated determinant of physical health. Current Opinion in Psychology. 2022;43:232–7. 34438331 10.1016/j.copsyc.2021.07.012

[59] Cosco TD, Howse K, Brayne C. Healthy ageing, resilience and wellbeing. Epidemiol Psychiatr Sci. 2017;26(6):579–83. 28679453 10.1017/S2045796017000324 PMC6998987

[60] Drewelies J, Koffer RE, Ram N, Almeida DM, Gerstorf D. Control diversity: How across-domain control beliefs are associated with daily negative affect and differ with age. Psychol Aging. 2019;34(5):625–39. 31192626 10.1037/pag0000366 PMC6684347

[61] Gerstorf D, Drewelies J, Duezel S, Smith J, Wahl HW, Schilling OK, et al. Cohort differences in adult-life trajectories of internal and external control beliefs: A tale of more and better maintained internal control and fewer external constraints. Psychol Aging. 2019;34(8):1090–108. 31804114 10.1037/pag0000389

[62] Smith BW, Dalen J, Wiggins K, Tooley E, Christopher P, Bernard J. The brief resilience scale: Assessing the ability to bounce back. International Journal of Behavioral Medicine. 2008;15(3):194–200. 18696313 10.1080/10705500802222972

[63] infas. SOEP-Core– 2021: Personenfragebogen, Stichproben A-L3, M1-M2 + N-Q. SOEP Survey Papers 1195: Series A– Survey Instruments (Erhebungsinstrumente). Berlin; 2023. https://www.diw.de/documents/publikationen/73/diw_01.c.866868.de/diw_ssp1195.pdf.

[64] Stalvey BT, Owsley C, Sloane ME, Ball K. The Life Space Questionnaire: A Measure of the Extent of Mobility of Older Adults. J App Gerontol. 1999;18(4):460–78.

[65] Jagger C, Gillies C, Cambois E, Van Oyen H, Nusselder W, Robine JM. The Global Activity Limitation Index measured function and disability similarly across European countries. J Clin Epidemiol. 2010;63(8):892–9. 20171842 10.1016/j.jclinepi.2009.11.002

[66] Health and Retirement Study. Questionnaire on Your Everyday Life and Well-being: Univeristy of Michigan; 2022 [Available from: https://hrs.isr.umich.edu/sites/default/files/meta/2022/core/qnaire/2022_SAQ.pdf.

[67] Nicklett EJ, Lohman MC, Smith ML. Neighborhood Environment and Falls among Community-Dwelling Older Adults. Int J Environ Res Public Health. 2017;14(2):175. 10.3390/ijerph14020175 PMC5334729 28208598

[68] Sharifian N, Spivey BN, Zaheed AB, Zahodne LB. Psychological distress links perceived neighborhood characteristics to longitudinal trajectories of cognitive health in older adulthood. Soc Sci Med. 2020;258:113125. 32599413 10.1016/j.socscimed.2020.113125 PMC7363567

[69] Kim ES, Hawes AM, Smith J. Perceived neighbourhood social cohesion and myocardial infarction. J Epidemiol Community Health (1979-). 2014;68(11):1020–6. 10.1136/jech-2014-204009 PMC4600604 25135074

[70] Garner IW, Holland CA. Age-friendliness of living environments from the older person’s viewpoint: development of the Age-Friendly Environment Assessment Tool. Age and Ageing. 2019;49(2):193–8. 10.1093/ageing/afz146 31790132

[71] Chan A, Malhotra C, Malhotra R, Østbye T. Living arrangements, social networks and depressive symptoms among older men and women in Singapore. International Journal of Geriatric Psychiatry. 2011;26(6):630–9. 20677171 10.1002/gps.2574

[72] Stadler G, Chesaniuk M, Haering S, Roseman J, Straßburger VM, Martina S, et al. Diversified innovations in the health sciences: Proposal for a Diversity Minimal Item Set (DiMIS). Sustainable Chemistry and Pharmacy. 2023;33.

[73] Stanford SPARQ. MacArthur Scale of Subjective Social Status– Adult Version Stanford: Stanford SPARQ, Department of Psychology; 2000 [Available from: https://sparqtools.org/mobility-measure/macarthur-scale-of-subjective-social-status-adult-version/.

[74] Hoebel J, Müters S, Kuntz B, Lange C, Lampert T. Messung des subjektiven sozialen Status in der Gesundheitsforschung mit einer deutschen Version der MacArthur Scale. Bundesgesundheitsblatt - Gesundheitsforschung - Gesundheitsschutz. 2015;58(7):749–57. 10.1007/s00103-015-2166-x 25986532

[75] Jaeger J. Digit Symbol Substitution Test: The Case for Sensitivity Over Specificity in Neuropsychological Testing. J Clin Psychopharmacol. 2018;38(5):513–9. 30124583 10.1097/JCP.0000000000000941 PMC6291255

[76] Swindell WR, Cummings SR, Sanders JL, Caserotti P, Rosano C, Satterfield S, et al. Data mining identifies Digit Symbol Substitution Test score and serum cystatin C as dominant predictors of mortality in older men and women. Rejuvenation Res. 2012;15(4):405–13. 22607624 10.1089/rej.2011.1297 PMC3419847

